# Reliability of Serum Metabolites over a Two-Year Period: A Targeted Metabolomic Approach in Fasting and Non-Fasting Samples from EPIC

**DOI:** 10.1371/journal.pone.0135437

**Published:** 2015-08-14

**Authors:** Marion Carayol, Idlir Licaj, David Achaintre, Carlotta Sacerdote, Paolo Vineis, Timothy J. Key, N. Charlotte Onland Moret, Augustin Scalbert, Sabina Rinaldi, Pietro Ferrari

**Affiliations:** 1 International Agency for Research on Cancer, Lyon, France; 2 Institute of Community Medicine, Faculty of Health Sciences, University of Tromsø, Tromsø, Norway; 3 Unit of Cancer Epidemiology, AO Citta’ della Salute e della Scienza-University of Turin and Center for Cancer Prevention (CPO-Piemonte), Turin, Italy; 4 Human Genetics Foundation (HuGeF), Turin, Italy; 5 School of Public Health, Imperial College London, London, United Kingdom; 6 Cancer Epidemiology Unit, Nuffield Department of Population Health, University of Oxford, Oxford, United Kingdom; 7 Department of Epidemiology, Julius Center for Health Sciences and Primary Care, University Medical Center, Utrecht, The Netherlands; University of Modena & Reggio Emilia, ITALY

## Abstract

**Objective:**

Although metabolic profiles have been associated with chronic disease risk, lack of temporal stability of metabolite levels could limit their use in epidemiological investigations. The present study aims to evaluate the reliability over a two-year period of 158 metabolites and compare reliability over time in fasting and non-fasting serum samples.

**Methods:**

Metabolites were measured with the AbsolueIDQp180 kit (Biocrates, Innsbruck, Austria) by mass spectrometry and included acylcarnitines, amino acids, biogenic amines, hexoses, phosphatidylcholines and sphingomyelins. Measurements were performed on repeat serum samples collected two years apart in 27 fasting men from Turin, Italy, and 39 non-fasting women from Utrecht, The Netherlands, all participating in the European Prospective Investigation into Cancer and Nutrition (EPIC) study. Reproducibility was assessed by estimating intraclass correlation coefficients (ICCs) in multivariable mixed models.

**Results:**

In fasting samples, a median ICC of 0.70 was observed. ICC values were <0.50 for 48% of amino acids, 27% of acylcarnitines, 18% of lysophosphatidylcholines and 4% of phosphatidylcholines. In non-fasting samples, the median ICC was 0.54. ICC values were <0.50 for 71% of acylcarnitines, 48% of amino acids, 44% of biogenic amines, 36% of sphingomyelins, 34% of phosphatidylcholines and 33% of lysophosphatidylcholines. Overall, reproducibility was lower in non-fasting as compared to fasting samples, with a statistically significant difference for 19–36% of acylcarnitines, phosphatidylcholines and sphingomyelins.

**Conclusion:**

A single measurement per individual may be sufficient for the study of 73% and 52% of the metabolites showing ICCs >0.50 in fasting and non-fasting samples, respectively. ICCs were higher in fasting samples that are preferable to non-fasting.

## Introduction

Advancement of analytical technologies has made high-throughput metabolic profiling of biological specimens possible [1]. Mass spectrometry techniques have been increasingly used to characterize the human metabolome [1,2] and the complex metabolic effects of nutrients or foods on chronic diseases in large-scale epidemiological studies [3–7], mainly because of their sensitivity and selectivity. However, most of the epidemiological studies rely on a single measurement of the metabolome. An eventual lack of temporal stability of the metabolome may bias relative risks based on a single measurement towards the null and could limit the significance of the findings [8–10].

Reliability studies evaluate the reproducibility of measurements taken on the same individuals at separate times. Reliability over time improves as the proportion of the total variance due to the between-subject variance increases. The reliability over time of metabolic profiles has recently been investigated in metabolites measured in blood samples [11–15]. Overall, fair to good reliability was reported, particularly in amino acids, hexoses, phosphatidylcholines, and sphingomyelins [11,12,15], except some acylcarnitines compounds that may yield poorer reliability [11,12]. Those reliability studies focused on assessing the temporal stability of metabolites measured in fasting blood samples. However, some epidemiological studies investigating metabolomics-disease links have used blood samples from non-fasting participants [2,16,17]. As food intake influences the metabolome [18], it has been hypothesized that the reliability was weaker in non-fasting than in fasting samples. This motivates the need of a study to compare the reliability of fasting and non-fasting samples.

The present study aims (1) to evaluate the reliability of metabolic profiling measurements over time in serum samples collected on average 2 years apart in subjects participating in the European Prospective Investigation into Cancer and Nutrition (EPIC) study, separately for fasting and non-fasting measures, and (2) to statistically compare reliability over time in fasting and non-fasting subgroups.

## Materials and Methods

### Study population and blood collection

EPIC is a large European cohort involving more than 521,000 subjects from 23 centers in 10 European countries [19]. All participants gave written informed consent to the EPIC study and the Ethical Committee of the International Agency of Research on Cancer specifically approved the present study. The EPIC cohort has at present an average follow-up of about 11 years. All participants completed questionnaires on diet, lifestyle, and medical history. In addition, for about 80% of EPIC participants a blood sample was collected at recruitment [19]. The EPIC cohort is particularly suitable to carry out epidemiological investigations on the link between metabolomics and risk of cancer. The current study was based on a sub-sample of participants who gave blood samples at two different time points: 39 non-fasting women from Utrecht (the Netherlands) and 27 fasting men from Turin (Italy). All the participants included in the present study were free of disease (diabetes, stroke, heart and overall cancer) at baseline. Although fasting and non-fasting individuals were different regarding gender and origin, this specific population was chosen as information about fasting conditions in these EPIC participants were known and consistent at both time points.

In Utrecht, women aged 50–65 years old, with no history of diabetes, cardiovascular disease or cancer, were invited to participate in a breast cancer screening from 1993 to 1999. A second examination with blood collection was carried out in a subsample of the cohort. Among all women attending this second examination, 39 who gave non-fasting blood samples in both occasions at 2.4 years interval (5^th^-95^th^: 2.2–3.9) were selected. In Turin, blood donors men aged 35–64 years were recruited from 1993 to 1998. A second blood drawn was taken in 39 men of which 27 who gave fasting blood samples in both occasions at 1.9 years interval (5^th^-95^th^: 1.0–2.8) were selected.

All the collected specimens were treated and banked with the same protocol as previously detailed for the whole cohort [20,21]. In short, blood samples were drawn in tubes without any anticoagulant (serum fraction), stored at +4°C until centrifugation (4,000 rpm for 20 minutes), and then stored in liquid nitrogen at -196°C.

### Biomarker analyses

Concentrations in serum samples for 158 endogenous metabolites were determined at IARC by ultra-performance liquid chromatography (LC) (1290 Series HPLC; Agilent, Les Ulis, France) hyphenated to a tandem mass spectrometer (MS/MS) (QTrap 5500; AB Sciex, Les Ulis, France) using the AbsoluteIDQ p180 kit (Biocrates, Innsbruck, Austria). The AbsoluteIDQ p180 Kit (Biocrates, Innsbruck, Austria) is a combined flow injection (FIA) and LC-MS/MS assay. The assay quantifies up to 188 metabolites from five analyte groups: acylcarnitines, amino acids, biogenic amines, hexoses (sum of hexoses), phosphatidylcholines (PCs), and sphingomyelins (SMs). The method combines the derivatization with 5% Phenylisothiocyanate reagent and extraction of analytes using 5mM ammonium acetate in methanol and the selective mass spectrometrical detection using MRM pairs. Isotope-labeled internal standards are partially integrated in the kit plate filter for metabolite quantification. Samples were analyzed using an LC/MS (QTrap 5500; AB Sciex, Les Ulis,France) method (for analysis of amino acids and biogenic amines) followed by FIA-MS (analysis of lipids, acylcarnitines and hexose). The limit of detection for the individual metabolites was set to three times the values of the bufferonly- containing samples. The MetIQ software package (BIOCRATES) allows an automation of the assay workflow, from sample registration to data processing. The AbsoluteIDQ p180 Kit validated by the manufacturer according to the Food and Drug Administration guideline ‘Guidance for industry–Bioanalytical Method Validation, May 2011’. For analytical specifications, refer to the AbsoluteIDQ p180 Kit manuals.

Measurements were made following the procedure recommended by the kit manufacturer. The kit was first tested on EPIC orphan samples, and 158 metabolites out of the proposed 188 were analytically validated. Repeated samples from the same subject were measured within the same analytical batch. Two different quality controls were inserted in each analytical batch. Intra- and inter-batch coefficients of variation ranged from 4% to 15% for the vast majority of analytes. Overall, 4.9% and 1.8% of total values were below the limit of detection (LOD) and the limit of quantification (LOQ), respectively. These values were imputed as LOD and LOQ values, respectively. However, reliability was not assessed when the percentage of values below LOD or LOQ for a given metabolite was greater than 50%, separately in fasting and non-fasting samples, which allowed 140 metabolites to be assessed for reliability.

### Variables definition

Samples were defined as fasting samples if time since last food or drink at blood collection was more than six hours. Samples that have been collected less than six hours since last food or drink were considered as non-fasting samples.

Body weight and height were measured at baseline according to standardized procedures previously described [22]. Briefly weight was measured to the nearest 0.1kg and height was measured to the nearest 0.1, 0.5, or 1.0 cm depending on the center, without shoes. The body mass index (BMI) was calculated as body weight in kilograms divided by squared height in meters (kg/m^2^).

Occupational, recreational and household physical activities were recorded by the EPIC physical activity questionnaire previously described [23]. Total physical activity was estimated as a categorical index (inactive, moderately inactive, moderately active, active) by cross-tabulation of the level of occupational activity (nonworker, sedentary, standing, manual, heavy manual and unknown) with combined recreational and household activities (in quartiles of MET-hours/week).

Other variables such as age (years), smoking status (current, former, never) and time difference between the two blood drawn (years), were also considered in analysis.

### Statistical analyses

Concentration levels of biomarkers were log-transformed to approximate normality. Estimates of the intraclass correlation coefficient (ICC) for each metabolite were computed to assess reliability over time [24] as the ratio of between-subject variance component over total variability in mixed models. For each metabolite, the measurement y_ijk_, with i = 1,..,n_k_ indexing study participant, j = 1,2 blood collection occasion and k = 1,2 center, is modelled through a random-effect model as

$$Y=\alpha +\;\beta \cdot X_{jk}+t_{j\left(k\right)}+e_{ij\left(k\right),}$$

$$t_{j(k)}∼N\left(0,\sigma_{Bk}^{2}\right)\;and\;e_{ij\left(k\right)}∼N\left(0,\sigma_{Wk}^{2}\right)$$

with *α* expressing the overall intercept, and *β* a vector of fixed-effect coefficients to capture the role of confounding variables, notably participants’ age, BMI and the time difference between sample collections. The terms *t*_*i(k)*_ and *e*_*ij(k)*_ are center-specific random-effects study participants and residuals, respectively, whose variance estimates, *σ* ^*2*^_*Bk*_ and *σ* ^*2*^_*Wk*_, estimate between- and within-subject variability. In this way the center-specific ICC estimates are computed as $\frac{\sigma_{Bk}^{2}}{\sigma_{Bk}^{2}+\sigma_{Wk}^{2}}$. The random-effects models were fitted by using the SAS PROC MIXED procedure which uses a restricted maximum likelihood function for parameter estimation.

The reproducibility of metabolites was assessed according to fasting status, i.e. comparing fasting samples in Turin and non-fasting samples in Utrecht. In order to control for differences of age, BMI and the time difference between sample collections across the two centers, these data were included as fixed effects in the models, whereby smoking status and physical activity had no virtually influence over variance of any of the metabolites. Reliability was considered as excellent with ICCs≥0.75, good with 0.50≤ICCs≤0.74, weak with ICCs<0.50.

The difference in ICC estimates according to fasting status was tested for statistical significance using a bootstrap sampling scheme [25]. A total of 300 repetitions provided sufficiently stable estimates. For each metabolite, P-values were obtained by comparing the ratio of the mean of the ICC difference between fasting and non-fasting samples over the standard deviation of the bootstrap distribution to a standardized normal distribution.

All statistical analyses were performed using SAS 9.2 statistical software (SAS Institute Inc, 2002).

## Results

At study entry, compared to women from Utrecht, men from Turin were younger, more frequently current smokers and less physically active, as reported in Table 1 **.**

**Table 1:** Characteristics of Turin and Utrecht EPIC participants at study entry.

	Turin (n = 27)	Utrecht (n = 39)
	Fasting men	Non-fasting women
**Age at baseline collection (years)** ^**a**^	47.4 (39.2–60.0)	60.1 (52.8–67.0)
**Smoking, n (%)**		
** Never**	6 (22%)	14 (36%)
** Former**	7 (26%)	17 (44%)
** Current**	13 (48%)	7 (18%)
** Unknown**	1 (4%)	1 (3%)
**Physical activity, n (%)**		
** Inactive**	3 (11%)	4 (10%)
** Moderately inactive**	5 (19%)	8 (21%)
** Moderately active**	10 (37%)	11 (28%)
** Active**	8 (30%)	15 (38%)
** Unknown**	1 (4%)	1 (3%)
**BMI (kg/m** ^**2**^ **)** ^**a**^	25.5 (21.1–29.6)	24.0 (19.2–32.3)
**Time difference (years)** ^**a**^	1.9 (1.0–2.8)	2.4 (2.2–3.9)

^a^For continuous variables the mean value and the 5^th^-95^th^ percentiles are provided.

### Reliability over time

**Fig 1:**
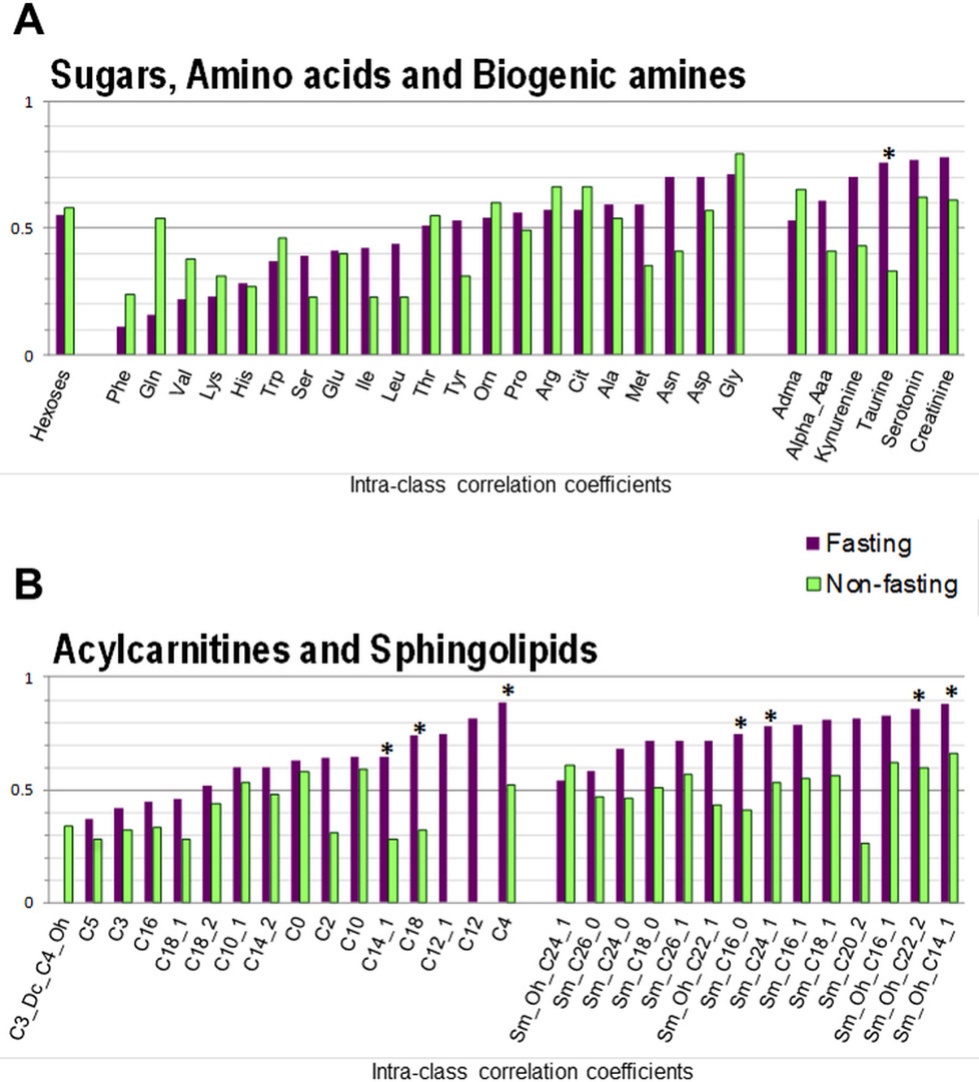
Intra-class correlation coefficients (ICCs) of serum sugars, amino acids, biogenic amines (A), acylcarnitines and sphingolipids (B) targeted metabolites in 27 fasting men and 39 non-fasting women. *P-values < 0.05 for difference between fasting and non-fasting ICCs

**Fig 2:**
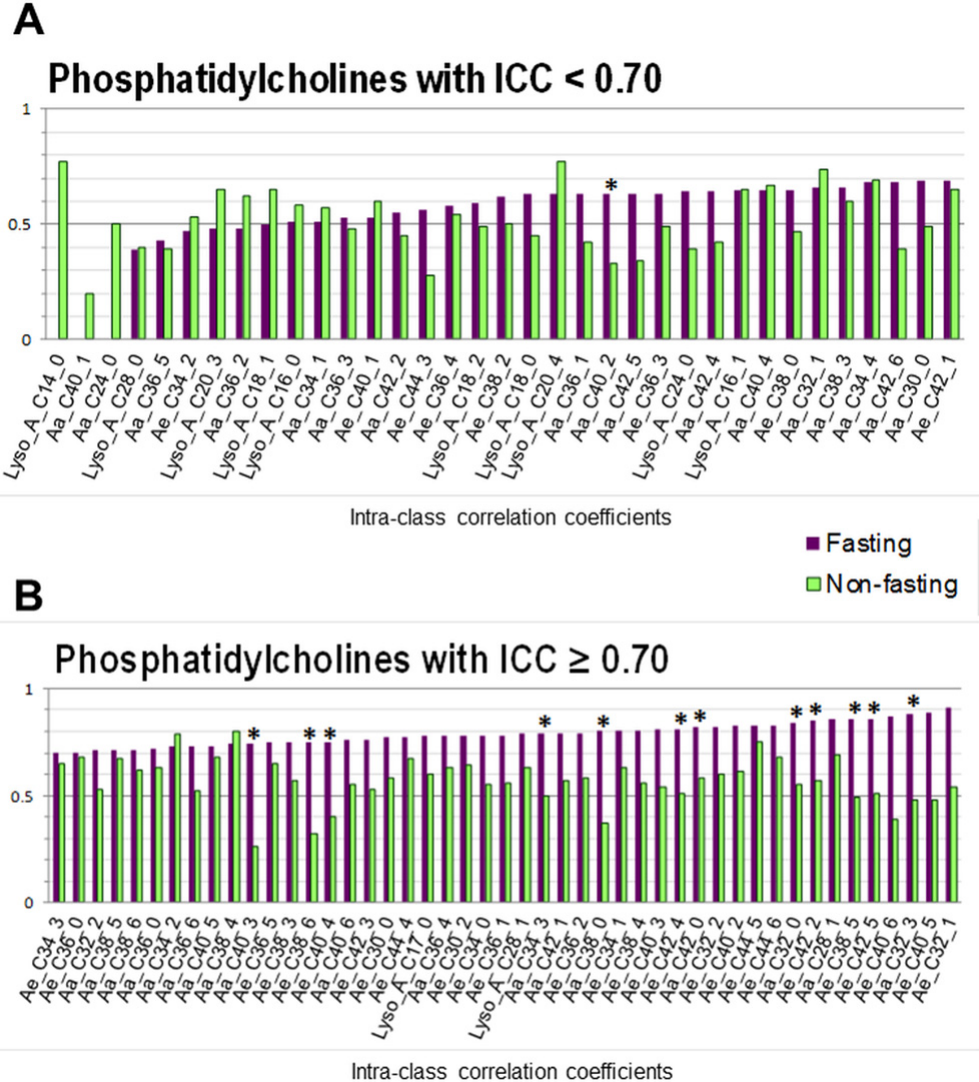
Intra-class correlation coefficients (ICCs) of serum targeted phosphatidylcholines in 27 fasting men and 39 non-fasting women according to their ICC values: ICCs < 0.70 (A); ICCs ≥ 0.70 (B). *P-values < 0.05 for difference between fasting and non-fasting ICCs

Mean levels, within- and between-variance and ICCs estimates of serum metabolites are reported in S1 Table and ICCs are represented in Figs 1 and 2 **.** In fasting samples from Turin, median ICC and ICC range were 0.62 (range: 0–0.89) for acylcarnitines, 0.51 (0.11–0.71) for amino acids, 0.77 (0.54–0.88) for SMs, 0.71 (0.53–0.78) for biogenic amines, and 0.55 for hexoses (Fig 1), and 0.63 (0.39–0.79) for lysoPCs and 0.74 (0.43–0.91) for PCs (Fig 2). Out of 140 metabolites, ICC values were higher than 0.70 for 58 metabolites (41%), and higher than 0.50 for 103 metabolites (74%). None of the hexoses, biogenic amines or SMs compounds showed ICCs<0.5. Among other groups, 48% of amino acids, 27% of acylcarnitines, 18% of lysoPCs and 4% of PCs compounds had ICCs lower than 0.50.

In non-fasting samples from Utrecht, median ICC and ICC range were respectively, 0.34 (range: 0.28–0.59) for acylcarnitines, 0.56 (0.23–0.79) for amino acids, 0.54 (0.26–0.66) for SMs, 0.46 (0.06–0.73) for biogenic amines, and 0.58 for hexoses (Fig 1), and 0.62 (0.39–0.77) for lysoPCs and 0.55 (0.20–0.79) for PCs (Fig 2). Out of 140 metabolites, ICC values were higher than 0.7 for 12 metabolites (8%), and higher than 0.5 for 73 metabolites (52%). ICCs<0.5 were seen in 71% of acylcarnitines, 48% of amino acids, 44% of biogenic amines, 36% of SMs, 34% of PCs and 33% of lysoPCs compounds.

In both fasting and non-fasting samples, weak reliability over time was observed in some amino acids, with values ranging from 0.11 to 0.46 for phenylalanine, glutamine, glutamic acid, valine, lysine, histidine, serine, leucine and isoleucine, and a few acylcarnitines compounds, with values lower than 0.46 for C3_Dc_C4_Oh, C5, C3, C16 and C18:1.

### Comparison of reliability of fasting vs non-fasting samples

Overall, reliability over time was lower when analyzing non-fasting samples (median ICC: 0.54 with 5^th^ -95^th^ percentile values ranging from 0.26 to 0.75) than fasting samples (0.70; 0.37–0.86). This was particularly apparent for acylcarnitines, PCs and SMs (ICC is lower in non-fasting samples for the majority of them). Reliability of amino acids, biogenic amines, hexoses and lysoPC was overall not different according to fasting status (Figs 1 & 2). ICCs were significantly lower in non-fasting *vs*. fasting samples in 36% of SMs, 19% of acylcarnitines, 20% of PCs, 17% of biogenic amines, and 0.05% of amino acids (S1 Table).

## Discussion

Most of the 140 investigated metabolites showed good reliability over time in serum samples collected on average 2 years apart in participants from the EPIC study. Reliability over time was overall good for non-fasting samples, with a median ICC value equal to 0.54, but metabolites analyzed from fasting samples generally exhibited larger reproducibility (ICC_median_ equal to 0.70). Although reproducibility of amino acids, biogenic amines, hexoses and lysoPC did not generally differ according to fasting status, acylcarnitines, PCs and SMs were generally less stable over time for non-fasting compared to fasting samples. It may be noticed that weak reliability over time was observed for some amino acids and acylcarnitines compounds for both fasting and non-fasting samples.

Previous studies have already investigated the reproducibility of endogenous metabolites using the same assay as we used (Biocrates kit) over short time periods in fasting samples [11,12,15]. In 22 healthy German volunteers who donated 3 fasting samples each day, Breier *et al*. found good reproducibility of most metabolites over a short time period of 3 consecutive days (ICC_median_ equal to 0.66), except for long chain and unsaturated acylcarnitines [11]. In 100 EPIC German subjects who gave 2 fasting samples 4 months apart, Floegel *et al*. reported an overall median ICC of 0.57, with median ICC estimates equal to 0.58 for amino acids, 0.58 for PCs, 0.66 for SMs, 0.76 for hexoses and 0.45 for acylcarnitines [12]. In line with previous findings [11], poor reproducibility in hydroxy- (0.11 ≤ ICCs ≤ 0.45) and monounsaturated acylcarnitines (0.09 ≤ ICCs ≤ 0.63) was mainly attributed to low concentrations [11,12]. In the study by Yu *et al*. involving 83 repeated fasting samples, most of hydroxy- and unsaturated acylcarnitines were excluded from the analyses because of low concentrations or poor reliability [15]. Among those analytically validated with concentrations higher than LOQ/LOD (16 out of 55), the present study exhibited good reliability of acylcarnitines with a median ICC of 0.62 in fasting samples and good reliability of monounsaturated acylcarnitines (0.46 ≤ ICCs ≤ 0.75). Reproducibility of these 16 acylcarnitine compounds was particularly affected by fasting status, with a median ICC dropping to 0.34 in non-fasting samples. As acylcarnitines occur in the process of fatty acid translocation into the inner mitochondrial membrane for β-oxidation, blood acylcarnitine concentrations reflect the substrate flux through β-oxidation, and then could be particularly affected by the composition and the time since the last meal. The weak reproducibility of phenylalanine, glutamine, glutamic acid, valine, lysine, histidine, serine, leucine and isoleucine amino acids in our study did not confirm previous findings. Amino acids measured in fasting samples generally showed good reproducibility, ICCs ranging from 0.41 to 0.84 in previous studies [11,12]. Amino acids reproducibility was not particularly influenced by fasting status, possibly reflecting the tight genetic regulation of amino acids homeostasis [26].

The main limitation of the present study was the different center origin and gender of fasting and non-fasting samples that led to the comparison of individuals with general characteristics differences which may well have driven part of the difference in reliability. To partially correct for differences in participants’ age, BMI and the time difference between sample collections, a combined mixed model adjusted for these factors was employed. To our knowledge, reproducibility studies on metabolomics profiling have not investigated the effects of gender or country origin on reliability. Yet, the effect of gender on metabolites concentrations seems to be limited as it was estimated to account for 7.3% of variability [14]. The present study included subjects from general population (blood donors and women participating to a breast cancer screening) who were free of disease (diabetes, stroke, heart and overall cancer) at baseline. However, there is the lack of information on health status of subjects at the second time point. If participants had developed chronic conditions during the 2-year follow-up, the within-person variability would increase, leading to underestimation of reliability over time. Only two time points were available in the EPIC cohort for the assessment of metabolites reliability. To study the influence of the number of time points on reliability estimates, Sampson *et al*. compared ICCs of metabolites calculated from two time points one year apart to those estimated from three time points four years apart [14], and the authors found similar distribution of ICCs. As our study sample was limited in size, no adjustment for multiple testing was adopted for the comparison of fasting and non-fasting ICC estimates. This strategy was chosen in order to avoid non detection of differences between fasting and non-fasting ICC estimates, as the number of metabolites whose reliability was differential with respect to fasting status would have diminished drastically.

The main strength of our study was that we assessed reliability in a wide spectrum of metabolites including different classes of compounds with a validated high-throughput technique that can be applied to future metabolome studies. In particular, our results are likely to be generalizable for the whole EPIC cohort where metabolome measurements have been undertaken. Another strength is that our results showed overall good reliability over a 2-year interval which represents a longer time interval compared to previous investigations involving the same metabolites spectrum [11,12,15]. Finally, to our knowledge, this is the first study that compares reliability in fasting and non-fasting samples for the metabolites measured in this assay (Biocrates kit).

## Conclusion

The present study showed good reproducibility for most of the Biocrates metabolites over a 2-year period, including PCs, SMs, biogenic amines, hexoses and most of the amino acids and acylcarnitines in fasting samples from healthy men from the EPIC cohort. More variability over time was observed in the 140 metabolites when measured in non-fasting samples. Our results indicate that a single measurement could be sufficient for hexoses and most of the amino acids, biogenic amines, SMs, and PCs in non-fasting samples, but not for most of the acylcarnitines. Fasting samples are preferable to non-fasting.

## Supporting Information

S1 Table Percentage of samples below the limit of detection (%<LOD), mean concentrations and their 95% confidence intervals (CIs), between- (B) and within-subject (W) variance components, intraclass correlation coefficients (ICC) of serum concentrations measured in 66 healthy subjects from two EPIC centers. **P-values for difference in fasting and non-fasting samples are also reported**.
(XLSX)
